# Following Natural Autoantibodies: Further Immunoserological Evidence Regarding Their Silent Plasticity and Engagement in Immune Activation

**DOI:** 10.3390/ijms241914961

**Published:** 2023-10-06

**Authors:** David Szinger, Timea Berki, Péter Németh, Szabina Erdo-Bonyar, Diana Simon, Ines Drenjančević, Senka Samardzic, Marija Zelić, Magdalena Sikora, Arlen Požgain, Katalin Böröcz

**Affiliations:** 1 Department of Immunology and Biotechnology, Clinical Center, Medical School, University of Pécs, 7624 Pécs, Hungary; szinger.david@pte.hu (D.S.);; 2 Department of Physiology and Immunology, Faculty of Medicine Osijek, Josip Juraj Strossmayer University of Osijek, 31000 Osijek, Croatia; idrenjancevic@mefos.hr; 3 Scientific Centre for Excellence for Personalized Health Care, Josip Juraj Strossmayer University of Osijek, 31000 Osijek, Croatia; 4 Department of Public Health, Teaching Institute of Public Health for The Osijek-Baranja County, 31000 Osijek, Croatia; 5 Department of Microbiology, Parasitology, and Clinical Laboratory Diagnostics, Medical Faculty of Osijek, Josip Juraj Strossmayer University of Osijek, 31000 Osijek, Croatia

**Keywords:** autoantibody, natural autoantibody, anti-viral antibody, ELISA, serology, MMR, SARS-CoV-2, IgG, vaccine, immunization, plasticity, immunological network

## Abstract

Contradictory reports are available on vaccine-associated hyperstimulation of the immune system, provoking the formation of pathological autoantibodies. Despite being interconnected within the same network, the role of the quieter, yet important non-pathological and natural autoantibodies (nAAbs) is less defined. We hypothesize that upon a prompt immunological trigger, physiological nAAbs also exhibit a moderate plasticity. We investigated their inducibility through aged and recent antigenic triggers. Anti-viral antibodies (anti-MMR n = 1739 and anti-SARS-CoV-2 IgG n = 330) and nAAbs (anti-citrate synthase IgG, IgM n = 1739) were measured by in-house and commercial ELISAs using Croatian (Osijek) anonymous samples with documented vaccination backgrounds. The results were subsequently compared for statistical evaluation. Interestingly, the IgM isotype nAAb showed a statistically significant connection with anti-MMR IgG seropositivity (*p* < 0.001 in all cases), while IgG isotype nAAb levels were elevated in association with anti-SARS CoV-2 specific seropositivity (*p* = 0.019) and in heterogeneous vaccine regimen recipients (unvaccinated controls vector/mRNA vaccines *p* = 0.002). Increasing evidence supports the interplay between immune activation and the dynamic expansion of nAAbs. Consequently, further questions may emerge regarding the ability of nAAbs silently shaping the effectiveness of immunization. We suggest re-evaluating the impact of nAAbs on the complex functioning of the immunological network.

## 1. Introduction

Despite increasing evidence supporting the overt dynamic adaptation capacity of autoantibodies (AAbs) in relation to immunological activation, attention has mainly been focused on the pathological AAb formation and the subsequent potential adverse consequences [1,2,3,4,5,6]. Natural antibodies are involved in the pathogenesis of autoimmunity, but only a minority of nAbs and nAAbs have pathogenic features [7,8,9]. A large majority of natural antibodies, so-called natural autoantibodies (nAAb), bind to (self) neo-epitopes, apoptotic, and necrotic cells. Such self-binding antibodies cannot be considered pathogenic; hence, they represent a separate moiety within the AAb compartment [7,8,9,10,11]. Compared to the pathological ones, these non-pathological or nAAbs are much less studied. Although they usually remain quiet, these fundamental participants in the immunological network feature some very important, yet unrecognized, physiological functions [9,12,13,14,15,16,17,18,19,20,21,22,23,24,25,26,27,28,29,30,31].

According to current knowledge, the term “natural antibodies” (nAAbs) refers to immunoglobulin molecules preexistent prior to antigen stimulation, originating mainly from B1-B and marginal zone B cells. Despite their recognized significance in innate immune defense as well as in the removal of altered cells and debris, their restricted immunological capacities are also reflected by their underappreciated significance in scientific research. These antibodies have moderate affinity, are typically poly-reactive, and their levels are physiological and thought to be relatively constant throughout life [9,17,18,19,20,21,22,23,24,25,26,27,28,29,30,31]. However, in the modern literature, there is increasing evidence of the moderate inducibility of the nAAb repertoire [13,14,15,16,32,33,34,35,36,37].

In contrast to the antigen-specific antibodies that are produced by mature B cells through somatic hypermutation in a T-cell dependent pathway in response to a foreign pathogenic challenge, nAAbs are presumed to be non-antigen-specific towards pathogens [23,31,38,39,40]. Natural IgM antibodies are assumed to have been selected during immune evolution for their contributions to critical immune regulatory and housekeeping properties [19]. They are generally considered poly-reactive, with low affinity and broad specificity to both foreign and self-antigens. IgM isotype nAAbs perform diverse homeostatic functions from injurious to protective, depending on their cellular and molecular environment [41]. Their protective functions, especially, are widely discussed in the scientific literature [10,19,36,41,42,43]. Yet, because of their more ancient and conserved origin, levels of IgM isotype nAAbs have been historically considered relatively constant throughout life [21,44,45,46].

On the other hand, the self-reactive repertoire of IgG is established within the first 2–4 years of life; it is highly homogenous among children and similar to that expressed by the IgG of healthy young and older adults, whereas the repertoire of IgG reactivity toward foreign and self-antigens is diverse and dependent on the history of each individual’s immune system [28]. For a long time, the presence of self-reactive IgG autoantibodies in human sera was thought to represent a breakdown in central tolerance and was commonly associated with the development or onset of autoimmune diseases or conditions. It has been described that IgG autoantibodies are generally present and abundant in human sera, and their serum diversity is strongly influenced by age, gender, and the presence of specific diseases [47]. Contradictorily, it has also been suggested that serum IgG autoantibody profiles are unique to an individual and remarkably stable over time [47].

In the contemporary literature, an increasing number of communications are addressing the apparent controversy regarding the inducible versatility of the supposedly stabile and evolutionary conserved nAAb pool. According to newer findings in this field, it seems that pathogen-associated environmental triggers, as well as the host microbiome, can have a substantial impact on the makeup of the nAAb repertoire [48,49,50]. It is known that enhanced vaccination strategies combine primary and secondary vaccine components to achieve optimal bioavailability and bioactivity of target substances while exhibiting a sufficiently broad spectrum of immune stimulation [51]. Although vaccinations are primarily intended to prevent disease, it is possible that they may also have unintended effects on the body’s natural antibody repertoire [49]. We hypothesized that with a competent antigenic trigger, nAAbs may also display a moderate level of dynamic adaptability, detectable at the level of antibody titers. This theory is also supported by previously established scientific data [32,33,34,35]. Therefore, we used the immunoserological approach of addressing the scientific question of whether there is a quantifiable difference in the adaptation capacity of the nAAb pool in response to an aged antigenic trigger (childhood measles, mumps, rubella (MMR) vaccine(s) or infection with the natural virus) versus a relatively recent stimulation (provided by contemporary anti-SARS-CoV-2 vaccines).

Accordingly, we determined our research objectives as follows:

I. Is there an association between the aged, aforetime elicited anti-viral (measles, mumps, and rubella childhood vaccinations or natural infections) antibody levels and the nAAbs?
  In order to answer this question, firstly, we aimed to evaluate IgG antibody titers elicited by the historical measles, mumps, and rubella (MMR) vaccines (or the relevant viral pathogens). Similarly to our former seroepidemiological reports [33,52,53], we also intended to delineate potential gaps of humoral immunity. Secondly, we compared the specific MMR antigen-induced seropositivity results to nAAb (anti-citrate synthase: anti-CS) titers.
II. Is there an association between the relatively recent anti-SARS-CoV-2 IgG antigen-induced antibodies and the nAAbs?
  In order to answer this question, firstly, we aimed to evaluate IgG antibody titers elicited by the contemporary SARS-CoV-2 vaccines. Subsequently, our goal was to contrast the specific SARS-CoV-2 antigen-induced seropositivity results with the nAAb (anti-citrate synthase: anti-CS) titers.

## 2. Results

### 2.1. Relative Differences in Anti-MMR Seropositivity Ratios by Age Groups

**Figure 1:**
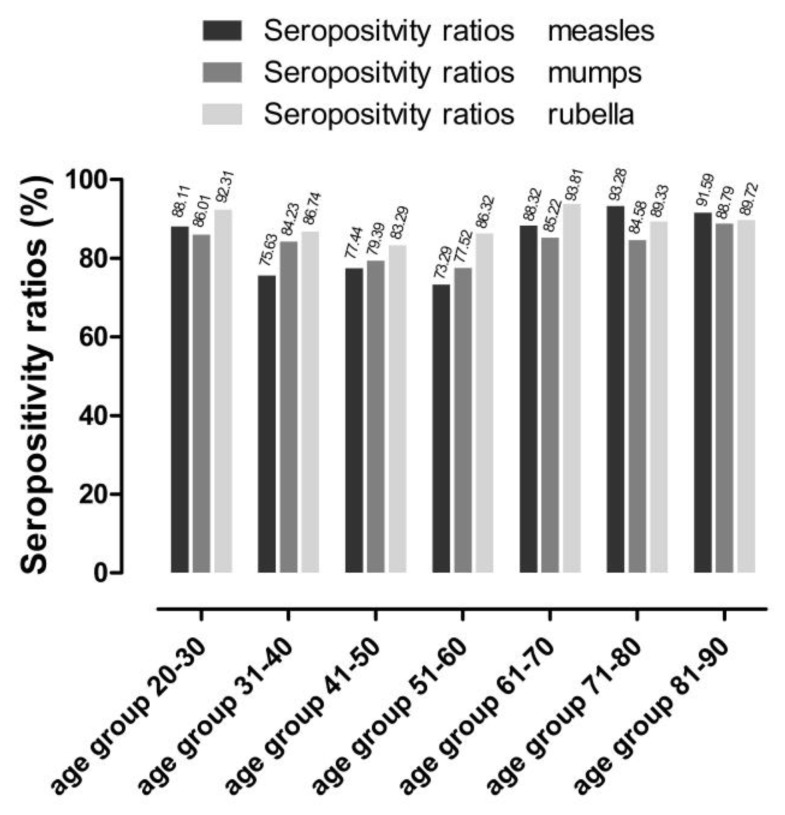
Measles, mumps, rubella (MMR) seropositivity ratios. N total = 1739, n measles = 1431, n mumps = 1438, n rubella = 1533. (For detailed age group numbers, please see Supplementary Table S1). The lowest seropositivity ratios were detectable in the age groups of 31–40, 41–50, and 51–60 (highlighted with red arrows). The herd immunity threshold (HIT) values were as follows: HIT measles = 92–95%, HIT mumps = 85–90%, HIT rubella = 83–86%.

In accordance with previous findings [53,54,55,56], in the recently tested Croatian samples, insufficiencies have been found in anti-MMR (and especially anti-measles) humoral protection (Figure 1). Focusing on anti-measles antibody titers, being the most concerning contributor [11,53,55,56,57,58,59,60,61,62,63,64,65,66] of the anti- MMR humoral immunity triad, the seropositivity ratios calculated based on circulating IgG antibody titers (number of positive samples/number of all samples ×100) were the most critical in the following age groups: 31–40 years, 41–50 years, and 51–60 years. Altogether, the findings illustrated herein can be considered suboptimal, as far as humoral antibody titers are considered “correlates of protection” [67,68,69,70,71]. To maintain stable anti-measles herd immunity, at least 92–95% of immunization coverage (in coexistence with adequate seroconversion) would be required [57,59,72,73].

### 2.2. Connection between nAAb (Anti-Citrate Synthase; Anti-CS) IgM Levels and Anti-Viral (MMR) Humoral IgG Levels

**Figure 2:**
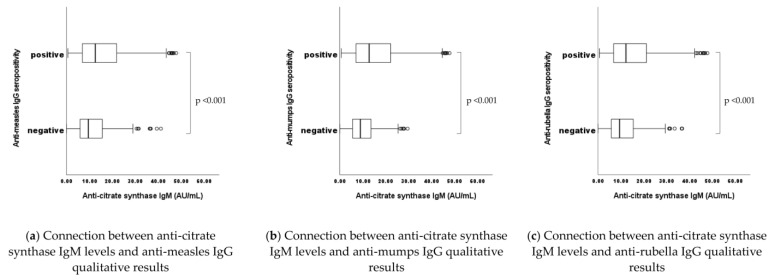
n total = 1739. (**a**) Measles: n negative = 308, n positive = 1431. (**b**) Mumps: n negative = 301, n positive = 1438. (**c**) Rubella: n negative = 206, n positive = 1533. Statistically significant connections have been found between anti-CS IgM levels and anti-viral (measles, mumps, rubella) IgG qualitative (positive, negative) results: in cases of adequate vaccine or infection-induced seropositivity, the natural antibody IgM levels also proved to be significantly higher (*p* < 0.001 for measles, mumps, and rubella).

When analyzing the effects of an aged antigenic trigger (MMR vaccines or natural infections with measles, mumps, or rubella) in terms of potential synergy between virus-specific antibodies and “adventitious” nAAbs (Figure 2), interestingly, statistically significant connections have been revealed in connection with the IgM isotype nAAbs. In case of viral antigen-triggered, anti-MMR seropositivity, the natural antibody IgM levels also proved to be significantly higher (*p* = 0.001 for measles, mumps, and rubella). Although they were measured and evaluated simultaneously, no statistically significant connections have been found between anti-CS IgG levels and anti-viral (measles, mumps, rubella) IgG qualitative (positive, negative) results.

### 2.3. Relative Differences in Anti-SARS-CoV-2 Specific Seropositivity Ratios by Age Groups

**Figure 3:**
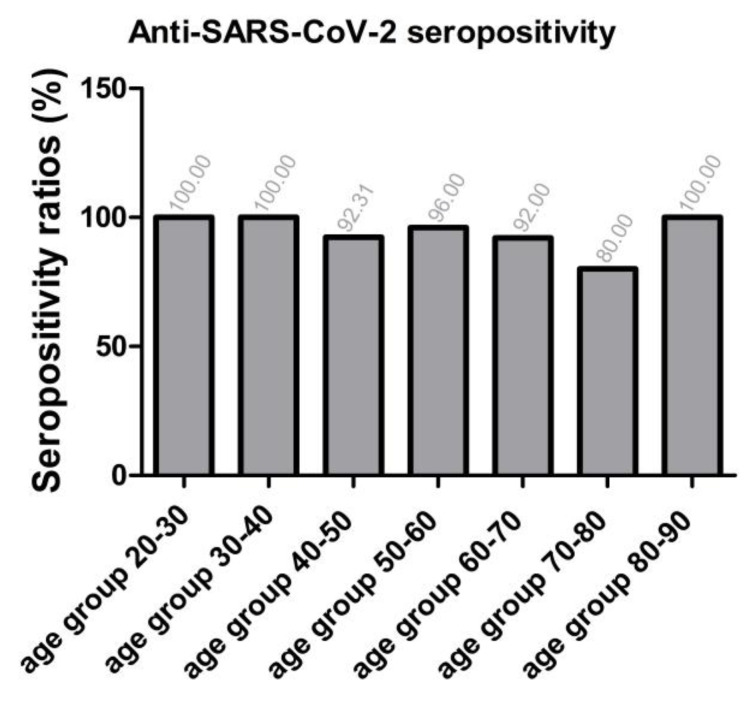
Anti-SARS-CoV-2 IgG seropositivity ratios, n total = 237, only vaccinated individuals. Sample numbers: n mRNA = 170, n Adenoviral vector = 25, n mRNA + adenoviral vector = 42. Sample numbers according to age groups: n 21–30 y = 21, n 31–40 y = 30, n 41–50 y = 26, n 51–60 y = 50, n 61–70 y = 50, n 71–80 y = 30, n 81–90 y = 22. (For detailed seropositive sample numbers per age group, please see Supplementary Table S2). Red bars show results calculated using the cut-off value as per manufacturer’s instructions: antibody titers ≥ 11 RU/mL have been considered ‘seropositive’.

As shown in Figure 3, in terms of anti-SARS-CoV-2 IgG seropositivity ratios (without differentiation between vaccines), the lowest seropositivity ratio (number of positive samples/number of all samples ×100) was found in the age group of 70- to 80-year-old individuals. Besides this latter group falling into the equivocal range, all clusters showed sufficiently high [74] seropositivity ratios of ≥80%.

### 2.4. Differences in Vaccine Response by Anti-SARS-CoV-2 Vaccines

**Figure 4:**
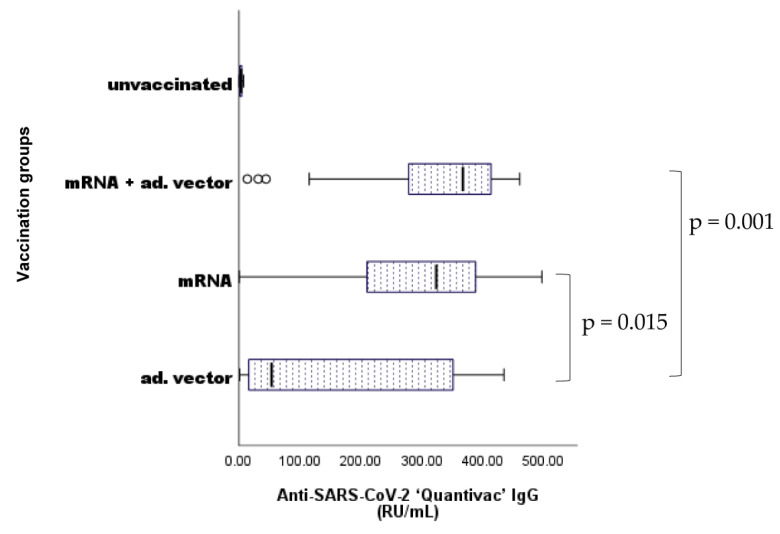
Differences in anti-SARS-CoV-2 IgG quantitative antibody titers between vaccination groups. Sample numbers: n unvaccinated = 93, n mRNA = 170, n adenoviral vector = 25, n mRNA + adenoviral vector = 42. n total = 330. Statistically significant differences have been found between the unvaccinated (control) group and all the other groups (*p* < 0.001); between the homologous adenoviral vector and the heterologous (mRNA/adenoviral vector vaccines) vaccination groups (*p* = 0.001); and between the mRNA and the adenoviral vector vaccine groups (*p* = 0.015).

We would like to emphasize that the primary focus of this article is not the comparative ranking of vaccine types or vaccination regimens based on their capacity to evoke humoral immune response that is relative to the correlates of protection [67,68,69,70,71]. Nevertheless, for the comparison between the ‘adventitious’ nAAbs and specific, viral antigen-triggered anti-SARS-CoV-2-specific ‘target’ antibodies, the measurement of anti-SARS-CoV-2 IgG titers was essential. As shown in Figure 4, we found significant differences between the unvaccinated (control) group and all the other groups (*p* < 0.001) (markers are not shown). Statistically significant differences have been found between the homologous adenoviral vector recipients and the heterologous vaccine regimen (mRNA/adenoviral vector vaccines) (*p* = 0.001), as well as between the mRNA and the adenoviral vector vaccine groups (*p* = 0.015).

Regarding the relationship between post-vaccination times (i.e., the number of days passed between sample taking and the last registered immunization) and anti-viral antibody titers, statistically significant inverse correlation was found only in the heterologous (mRNA/adenoviral vector vaccines) group: post-vaccination time/vaccine induced anti-SARS-CoV-2 IgG titers: spearman’s rho correlation coefficient < 0.001 (figure not shown).

### 2.5. Differences in nAAb (anti-CS) IgG Levels between Vaccination Groups

**Figure 5:**
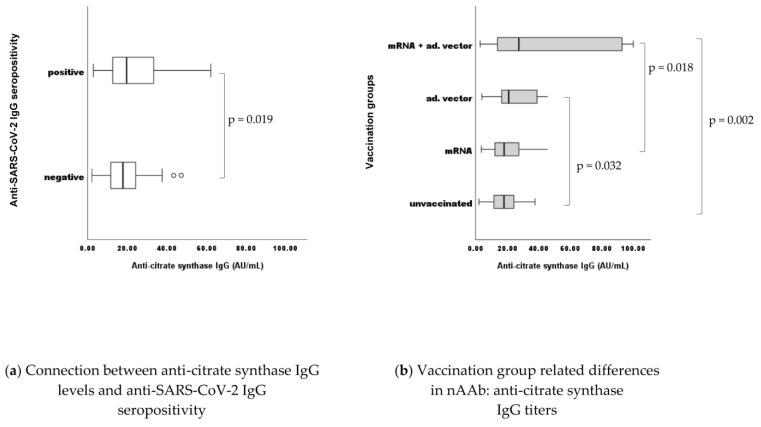
(**a**) n negative = 107, n positive = 222, n total = 330. Seropositivity evaluation was performed as per the manufacturer’s instructions (threshold: result ≥ 11 RU/mL). (**b**) Sample numbers: n unvaccinated = 93, n mRNA = 170, n adenoviral vector = 25, n mRNA + adenoviral vector = 42. n total = 330.

When analyzing the effects of a relatively recent antigenic trigger (SARS-CoV-2 vaccines) in terms of potential interplay between virus-specific antibodies and “adventitious” nAAbs (Figure 5a), statistically significant associations have been found in connection with the IgG isotype of nAAbs. In cases of anti-SARS-CoV-2 IgG seropositivity (positive result: titer ≥ 11 RU/mL as per manufacturer’s instructions), the nAAb levels (anti-CS IgG) also proved to be significantly higher (*p* = 0.019) (Figure 5a).The current finding aligns with data in the literature reporting their moderately inducible nature [47].

When analyzing the differences in nAAb (anti-CS) levels between the different vaccination groups and the unvaccinated controls (Figure 5b), again, we found statistically significant differences in terms of IgG isotype nAAbs: between the unvaccinated group and the adenoviral vector vaccine recipients (*p* = 0.032), the unvaccinated group and the heterologous vaccine regimen recipients (mRNA/adenoviral vector vaccines) (*p* = 0.002), and between the mRNA vaccine recipients and the heterologous group (*p* = 0.018). Interestingly, no statistical differences were detectable between the mRNA vaccine recipients and the unvaccinated individuals considering the nAAb (anti-CS Ig) levels. Although they were measured and evaluated in parallel, no statistically significant connections were found between anti-CS IgM levels and anti-viral (anti-SARS-CoV-2) IgG qualitative (positive, negative) results.

### 2.6. Global Summary of the Most Important Findings

**Figure 6:**
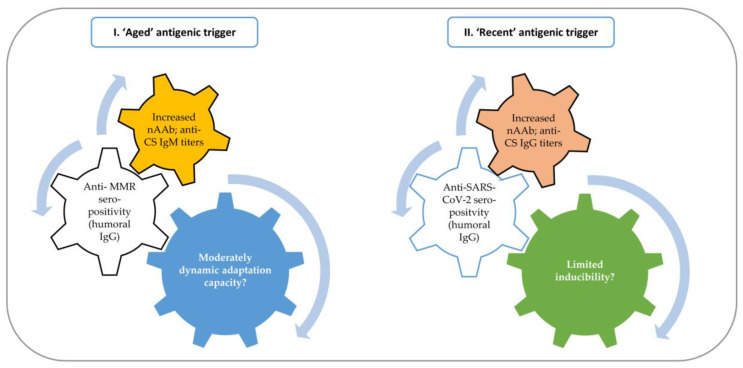
Global summary of the most important findings.

In order to provide an easier understanding of the examined immunological networking and potential associations between “target” antibodies and “off-target” or “adventitious” nAAbs, in the context of highly diversified epidemiological and purely immunoserological data, we summarize our most important results in Figure 6.

## 3. Discussion

Due to the evident burden of the COVID-19 pandemic on public health institutions, aggravated by the ongoing European refugee crisis, epidemiological concerns are re-emerging regarding the effectiveness of MMR vaccinations and population immunity levels [55,58,75]. Therefore, we updated our previous data regarding potentially inadequate humoral immunity levels in terms of anti-MMR IgG titers. In accordance with previous reports [34,53,54,56], present findings illustrate that potentially susceptible age groups might (also) be present in the Croatian population (Figure 1). As long as serum antibody concentrations are considered relative correlates of protection (46–50), this result underlines the critical significance of constant monitoring [55] of vaccine-induced humoral antibody titers.

Regarding anti-SARS-CoV-2-specific immunity, apart from the age group of 70–80-year-old individuals in the borderline range, in the age-weighed comparison, all clusters performed sufficiently well, with seropositivity ratios of ≥80%. Since our sample set is considered representative, the current data can be regarded as an estimation for population-level immunity. In the context of population immunity, these findings are meant to be within an acceptable range, since the herd immunity threshold value for SARS-CoV-2 variants of concern (B.1.1.7 “Alpha”) is usually cited around 80%, while for newer variants (B.1.617.2 “Delta”), it may be higher [74]. Nevertheless, it is important to highlight that, in cases of SARS-CoV-2 infections, the concepts of herd immunity, the “immunological protection”, and “reinfection risk”, are often debated [76,77]. The main barrier to achieving herd immunity is that SARS-CoV-2 is undergoing frequent mutations [78], a phenomenon which is also oftentimes aggravated by the human factor of vaccine hesitancy [76]. Therefore, the evaluation of population-level humoral immunity results, particularly with regard to estimated HIT values, is to be taken with prudence.

Considering the analysis according to vaccination groups, our results are consistent with data from previous studies [4]. Anti-SARS-CoV-2 antibody levels were lower after homologous adenoviral vector or mRNA vaccination compared to the heterologous vector/mRNA vaccine regimen recipients.

The above-described seroepidemiological analysis served as a cornerstone for understanding the dynamic interaction between nAAbs (anti-citrate synthase IgG, IgM) and viral-antigen-elicited (measles, mumps, rubella, SARS-CoV-2), promptly inducible antibodies. The main idea behind the current immunoserological study refers back to animal experiments: it has been described that exposure of laboratory rats to “wild-like” conditions can partially reconstitute the nAAb repertoire [48,49,50]. This practice of exposing laboratory animals to foreign antigens in order to manipulate their immune functions mimics the human medical practice of vaccination [48,49,50]. The empirical evidence provided by human immunization experience regarding the nonspecific effects (NSEs) of vaccines is also likely to be associated with “by-product” nAAbs [79,80,81,82].

Numerous accounts exist in the scientific literature regarding anti-SARS-CoV-2 vaccine-triggered hyperstimulation of the immune system. Some of these maintain that there is an elevated risk of vaccine-associated pathological auto-antibody formation [1,2,3], while others state that it is rather the natural infection being the major inducer of autoantibody formation [2,5,6], and that COVID-19 vaccines do not significantly foster the appearance of pathological autoantibodies commonly linked to the most prevalent autoimmune conditions [4]. Interestingly, the association between vaccination (or infection) and the non-pathological (natural) autoantibodies is much less studied [13,14,15,16].

When examining potential latent interactions in terms dynamic interplay between virus-specific “target” antibodies and “adventitious” nAAbs, upon an aged antigenic trigger like childhood MMR vaccines or potential earlier natural measles, mumps, or rubella infections, interestingly, statistically significant connections have been revealed between the anti-CS IgM levels and the viral-antigen-specific (measles, mumps, rubella) IgG qualitative (positive, negative) results. Although it is in accordance with previous findings [32,33,34], this result can be considered nonconformist, as IgM isotype nAAbs have been postulated to be more constant over time since their selection during immune evolution. IgM isotype nAAbs were primarily known for their immune-regulatory and housekeeping functions [10,19,32,41], rather than for their limitedly inducible nature.

When investigating the effects of a contemporary antigenic trigger (SARS-CoV-2 vaccines) in terms of potential associations between virus-specific “target” antibodies and “off-target” or “adventitious” nAAbs (Figure 5a), in accordance with data in the literature reporting their moderately inducible nature [47], statistically significant connections have been found in connection with the anti-CS IgG isotype nAAbs. The present result supports the current scientific opinion regarding the plasticity of IgG isotype nAAbs. Despite being considered relatively stable over time [8,11,29,41], IgG isotype nAAbs are supposed to be more prone to inducible dynamic changes influenced by age, gender, and pathogenic impacts [47]. Another curious finding in connection with IgG isotype nAAbs was that the heterologous vaccine regimen (mRNA/adenoviral vector vaccines) induced the highest antiviral IgG levels, also associated with the highest rate of nAAb formation. At the same time, our results suggest that the homologous regimen of mRNA vaccines did not induce an elevated nAAb formation: no statistically significant differences have been found compared to unvaccinated controls (Figure 5b).

## 4. Materials and Methods

### 4.1. Human Serum Samples

For measles, mumps, and rubella antigen-induced (MMR vaccine or natural infection) humoral antibody measurements, we evaluated a total of 1739 serum samples (Table 1) received from the Scientific Centre for Excellence for Personalized Health Care, Josip Juraj Strossmayer University of Osijek. These specimens were anonymous residual sera with known ages and COVID-19 vaccination histories (Table 1).

**Figure 7:**
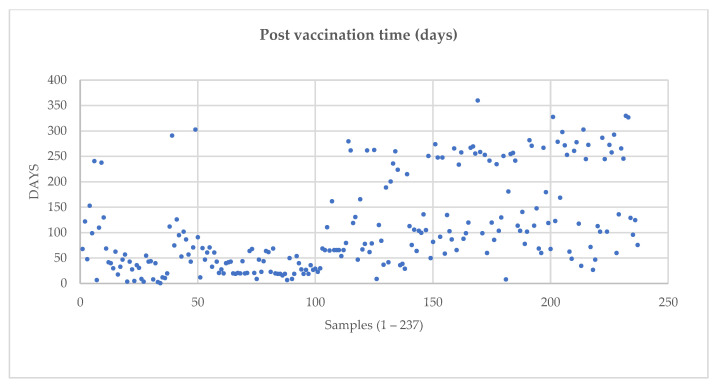
Distribution of post-vaccination times (days).

From this serum bank, we selected a multitude of samples representative of each age group, with the inclusion criterion of at least one documented anti-SARS-CoV-2 vaccination within one year. Due to limited research resources and high material purchase costs, not all of the serum banks could be screened for anti-SARS-CoV-2 IgG. Thus, 237 samples belonging to vaccinated individuals and 93 unvaccinated sera were selected (n total = 330) (Table 2) for evaluation. Vaccine-regimen-based subdivisions and post-vaccination times are represented in Figure 7 and Table 3.

For the investigation of potential connections between nAAb levels (anti-citrate synthase IgG, IgM) and immunization-induced humoral antibody titers, we performed anti-citrate synthase (CS) IgG, IgM measurements using the same serum bank.

### 4.2. Citrate Synthase (CS) IgG and IgM in-House ELISA Assays

As nAAbs, we used anti-citrate synthase (CS) antibodies; CS is a pacemaker enzyme in the Krebs cycle and is commonly used as a quantitative marker enzyme for the content of intact mitochondria [83,84]. As proven by the scientific literature [21,32,33,34,35,36,37,46,85,86], CS-specific autoantibodies can be considered a prominent example of nAAbs.

The same assay protocol already used for previous reports [33] was applied. Accordingly, 96-well polystyrene plates (NUNC) were coated with CS from porcine hearts (Sigma-Merck, Munich, Germany) in 0.1 M bicarbonate buffer, pH 9.6 [21]. Following this, the saturation of nonspecific binding sites with our alternative, combined blocking buffer (0.5% polyvinyl alcohol solution combined with bovine gelatin solution, at a ratio of 2:1) was performed at room temperature (RT) for 2 h. After being washed with PBS + 0.05% Tween 20 (washing buffer: WB), sera were diluted (1:100 in WB) and incubated for 50 min at 37 °C. The secondary antibodies were incubated at 37 °C for 45 min (horseradish peroxidase-conjugated antihuman IgG and IgM, polyclonal rabbit antihuman (Agilent-Dako Santa Clara, CA, USA)). TMB substrate solution (Sigma-Merck, Munich, Germany) was used to visualize the HRP enzymatic reaction, and the reaction was stopped by 1 M H_2_SO_4_. Reading was performed at λ = 450/620 nm using the BEP2000 Advanced automated system. The results are expressed in absorbance (OD) and in quantitative (standard-curve-based) results. For data comparison, the results were handled as continuous, non-normally distributed integers, and the alterations of the titers were considered. For the comparison of the virus-specific antigen-induced antibodies and nAAbs, both CS IgG and IgM isotypes were measured, but only the statistically significant positive connections are illustrated.

### 4.3. Anti-SARS-CoV-2 Quantivac ELISA (IgG)

Commercial SARS-CoV-2 Quantivac ELISA kits (EI 2606-9601-10 G: EUROIMMUN Medizinische Labordiagnostika AG, Lübeck, Germany) were applied as per the manufacturer’s standard. The ELISA assay provides quantitative in vitro determination of human antibodies of the immunoglobulin class IgG against SARS-CoV-2 in serum. The immunoassay supports the diagnosis of SARS-CoV-2 infection; moreover, serological data obtained using this kit can be applied to collect epidemiological data, as well as for antibody determination following vaccination with S1/RBD-based vaccines [87]. Reagent wells were coated with recombinant S1 domain of the spike protein of SARS-CoV-2. In the first reaction step, diluted samples (1:101) were incubated in the first wells. In the case of positive samples, specific IgG antibodies bound to the antigens. To detect bound antibodies, a second incubation was carried out using peroxidase enzyme-labeled anti-human IgG (enzyme conjugate), catalyzing a color reaction [87]. For test evaluation, the standard curve from which the concentration of antibodies in the samples (expressed in relative units: RU) could be calculated was obtained by point-to-point plotting of the extinction readings measured for the 6 calibration sera. The calibration sera had a linear correlation with the “First WHO International Standard for SARS-CoV-2”(NIBSC code 20/136), as stated in the Manufacturer’s Instructions for Use [87]. Euroimmun recommends quantitative result interpretation as follows: result < 8 RU/mL: negative, 11 RU/mL > result ≥ 8 RU/mL: borderline, result ≥ 11 RU/mL: positive [87].

### 4.4. Anti-Measles, Mumps, and Rubella (MMR) IgG in-House ELISA Assays

An assay protocol with the same assay execution guidelines thoroughly detailed in our previous publications [88,89] was applied. Briefly, the coating antigens were Bio-Rad PIP013 Measles virus, Edmonston strain (coating concentration: 2.8 µg/mL); Bio-Rad PIP014 Mumps virus, Enders strain (coating concentration: 3.0 µg/mL); and Bio-Rad PIP044 Rubella virus, HPV-77 strain (coating concentration: 0.4 µg/mL). The antigens were dissolved in ELISA Coating Buffer (Bio-Rad BUF030) and applied to 96-well plates overnight at 4–6 °C. Blocking was performed for ≥2 h, RT, with our in-house-developed, PVA-based blocking buffer. Standards: 3rd WHO International Standard for Anti-Measles (NIBSC code: 97/648), Anti-Mumps Quality Control Reagent Sample 1 (NIBSC code: 15/B664), and Anti-Rubella Immunoglobulin 1st WHO International Standard Human (NIBSC code: RUBI-1-94). Human serum samples were applied at a final dilution of 1:200 after non-specific background reduction (incubation followed by centrifugation) using a matrix-equalizing, mammalian-protein-containing buffer (IgM Reducing Assay Diluent- Bio-Rad BUF038) diluted in washing buffer at a ratio of 2:1. Washing steps: 5 times, automated. Uniform incubation times for primary, secondary antibody binding, and substrate reaction: 3 × 20 min, 37 °C. For the visualization of the immunological reaction, we used HRP-conjugated Dako polyclonal rabbit anti-human IgG (+TMB). Automated assay execution, photometric reading (λ = 450/620 nm), and quantitative result calculation (4-parametric fitting) were performed using a Siemens BEP 2000 Advance System. As an independent control and reference assay of our in-house ELISAs, commercial ELISA kits from EUROIMMUN were applied. For comparability reasons between tests, in-house ELISA assay cut-offs were harmonized with the reference tests.

### 4.5. Anti-Measles, Mumps, and Rubella Commercial ELISA Assays

Commercial kits from EUROIMMUN Medizinische Labordiagnostika AG (Lübeck, Germany) were used as validated controls, parallel to in-house assay measurements. Assay execution was performed as per the manufacturer‘s standard.

#### 4.5.1. Anti-Measles Virus ELISA (IgG) (EI 2610-9601 G)

The commercial kit was used to provide quantitative in vitro determination for IgG-class human antibodies against the measles virus in serum. The test kit contained microtiter strips, each with 8 break-off reagent wells coated with measles virus antigens (inactivated cell lysates of Vero cells infected with the “Edmonston” strain of the measles virus). In the first reaction step, diluted patient samples (1:101) were incubated in the wells. In the case of positive samples, specific IgG antibodies (also IgA and IgM) bound to the antigens. To detect the bound antibodies, a second incubation was carried out using an enzyme-labeled anti-human IgG (enzyme conjugate), catalyzing a color reaction. The controls of the Anti-Measles Virus ELISA (IgG) were calibrated using the 3rd international standard serum NIBSC 97/648 (anti-measles and anti-polio virus serum, National Institute for Biological Standards and Control, Hertfordshire, England).

Quantitative evaluation: the standard curve from which the concentration of antibodies in the patient samples could be taken was obtained by point-to-point plotting of the extinction values measured for the 4 calibrators against the corresponding units (linear/linear). Euroimmun recommends quantitative result interpretation as follows: result < 200 IU/L: negative, 275 IU/L > result ≥ 200 IU/L: borderline, result ≥ 275 IU/L: positive.

#### 4.5.2. Anti-Mumps Virus ELISA (IgG) (EI 2630-9601 G)

The commercial kit was used to provide quantitative in vitro determination for IgG class human antibodies against measles virus in serum. The test kit contained microtiter strips, each with 8 break-off reagent wells coated with mumps antigens (inactivated cell lysates of Vero cells infected with the “Enders” strain of the mumps virus). In the first reaction step, diluted patient samples (1:101) were incubated in the wells. In the case of positive samples, specific Ig antibodies bound to the antigens. To detect the bound antibodies, a second incubation was carried out using an enzyme-labeled anti-human IgG (enzyme conjugate), catalyzing a color reaction.

As no international reference serum exists for antibodies against the mumps virus, the calibration was performed in relative units (RU/mL).

Quantitative evaluation: The standard curve from which the concentration of antibodies in the patient samples could be taken was obtained by point-to-point plotting of the extinction readings measured for the 3 calibration sera against the corresponding units (linear/linear). Euroimmun recommends quantitative result interpretation as follows: result < 16 RU/mL: negative, 22 RU/mL > result ≥ 16 RU/mL: borderline, result ≥ 22 RU/mL: positive.

#### 4.5.3. Anti-Rubella Virus ELISA (IgG) (EI 2590-9601 G)

The commercial kit was used to provide quantitative in vitro determination for IgG class human antibodies against the measles virus in serum. The test kit contained microtiter strips, each with 8 break-off reagent wells coated with mumps antigens. (The antigen source was provided by inactivated cell lysates of Vero cells infected with the “HPV-77” strain of the rubella virus). In the first reaction step, diluted patient samples (1:101) were incubated in the wells. In the case of positive samples, specific Ig antibodies bound to the antigens. To detect the bound antibodies, a second incubation was carried out using an enzyme-labeled anti-human IgG (enzyme conjugate) catalyzing a color reaction. Calibration was performed in international units (I) using the international reference preparation NIBSC RUBI-1-94 (Anti-Rubella Serum, 1* International Standard for Anti-Rubella Immunoglobulin, Human, National Institute for Biological Standards and Control, Hertfordshire, England).

Quantitative evaluation: the standard curve from which the concentration of antibodies in the patient samples could be taken was obtained by point-to-point plotting of the extinction values measured for the 4 calibrators against the corresponding units (linear/linear). Euroimmun recommends quantitative result interpretation as follows: result < 16 RU/mL: negative, 8 IU/mL > result ≥ 11 IU/mL: borderline, result ≥ 11 IU/mL: positive.

### 4.6. Statistical Evaluation

For statistical evaluation (IBM SPSS), the Mann–Whitney U test was selected (α = 0.05). Natural autoantibody (nAAb) levels were treated as ordinal, non-normally distributed variables, while immunization-induced qualitative (positive, negative) results were designated as grouping parameters. Simple bar-chart-based seropositivity evaluations were represented using MS Excel 2016.

### 4.7. Experimental Design

**Figure 8:**
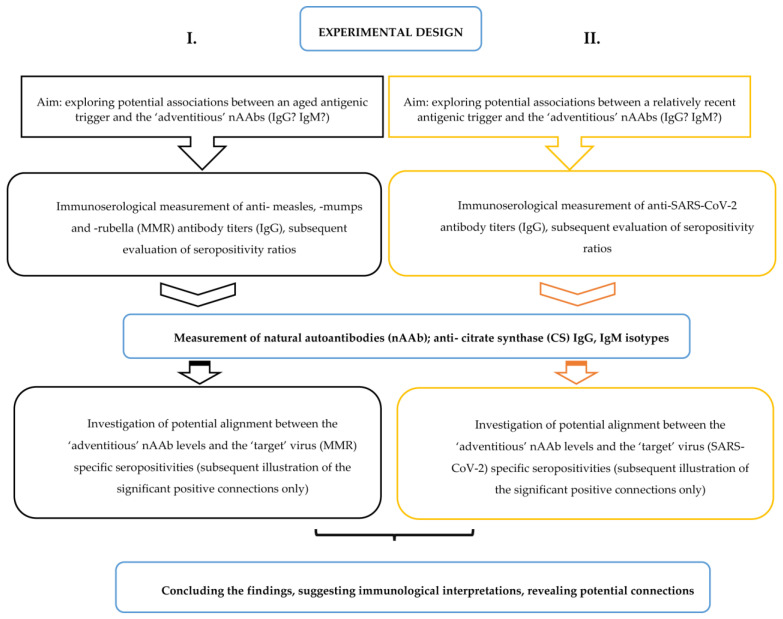
Schematic representation of the overall experimental design.

In order to provide a clearer understanding of the experimental design based on the research objectives already determined in Section 1, herein (Figure 8), we present a comprehensive sketch of the research methodology.

## 5. Conclusions

To summarize, herein we present epidemiological and immunoserological data on associations between virus-specific “target” antibodies and “off-target” or “adventitious” nAAbs (Figure 8). Our observations are supplemented by a growing body of literature investigating potential connections between immunization and the concurrent dynamic change in the nAAb repertoire [13,14,15,16,32,33,34,35,36,37]. Based on our current findings, in case of a contemporary antigenic trigger, for SARS-CoV-2 vaccines, in terms of potential associations between virus-specific antibodies and “adventitious” nAAbs, statistically significant associations have been found in connection with the IgG isotype of nAAbs. The result harmonizes with the literature data reporting their moderately inducible nature [8,11,29,41,47]. On the other hand, in cases of aged antigenic triggers: MMR childhood vaccinations (or natural infections), in terms of potential dynamic interplay between virus-specific “target” antibodies and “adventitious” nAAbs, statistically significant connections have been revealed in connection with the IgM isotype nAAbs. Although this finding is not without precursors [32,33,34], it can be considered paradoxical to the formerly established immunological dogma regarding the relatively constant nature and stable presence of nAAbs, set during the selection process of immune evolution [10,19,32,41]. The phenomenon investigated herein, delineated using immunoserological measurements, raises many questions regarding the silent, dynamic plasticity of natural nAAbs. Further investigations are needed in order to elucidate whether these antibody associations can be considered physiological; whether the isotype(s) involved in the natural autoantibody response are being influenced by dynamic changes or adaptability over time; and, lastly but maybe most importantly, whether there are potential feedback mechanisms on the effect of vaccination.

We maintain that only a comprehensive understanding of this type of immunological networking will be able to deploy the potential crosstalk between “target” antibodies and “adventitious” nAAbs. We propose the current paper as a reminder to immunologists that the existing insufficiencies of our knowledge must be amended in order to better understand the mechanisms behind heterologous “off-target” effects of vaccines, as well as to unravel the potential ensuing implications regarding individual vaccine responsiveness.

## 6. Implications of the Study

In cases of anti-MMR humoral antibody titer (IgG) assessment, the term “antigen triggered” is used with the deliberate purpose of avoiding distinction between purely vaccine-induced, infection-induced (measles, mumps, or rubella wild-type viruses), and “hybrid immunity” (developed through a combination of virus infection and vaccination) cases. Considering the lack of MMR vaccination- and/or disease-related data (specimens: anonymous residual sera with known age and COVID-19 vaccination history, as detailed in the Materials and Methods section), this can be considered a limitation of the study.

In the case of anti-SARS-CoV-2 humoral antibody titer (IgG) assessment and subsequent estimation of population-level humoral immunity result (extrapolated from a representative set of specimens), particularly with regard to estimated HIT values, the evaluation included herein should to be taken with circumspection. The reason for this implication is that although herd immunity is generally cited to be around 75–85%, it can be highly variable according to repeated mutations of the viral genome accompanied by viral evolution into new strains, sometimes even with enhanced severity [76,77,78]. Moreover, herd immunity also works as a function of the intensity of infection [77,90]. Herein, once more, we would like to emphasize that the primary focus of the present manuscript is not the ranking between vaccine types of regimens with regard to the anti-SARS-CoV-2 immunization programs.

In connection with the moderately inducible plasticity of the natural autoantibody anti-citrate synthase (IgG and IgM), the possibility of cross-epitope-associated induction of the immune system cannot be ruled out. Due to infeasibility, it has not been examined nor excluded. Therefore, it can be considered a limitation of this study. Nevertheless, in accordance with previous articles cited throughout the present manuscript [1,2,3,4,5,6,13,14,15,16,48,49,50,79,80,81,82], the primary focus of our investigation was to suggest an immunologically acceptable, maybe even thought-provoking explanation for the herein-detailed empirical phenomenon, detected and experimentally proven at the level of immunoserological measurements.

## Figures and Tables

**Table 1:** Age-group-based subdivision of samples used for anti-MMR IgG and anti-citrate synthase IgG/M screening.

Age Group	Total Number of Samples/Age Group
20–30 y	143
31–40 y	279
41–50 y	359
51–60 y	307
61–70 y	291
71–80 y	253
81–90 y	107
TOTAL	1739

**Table 2:** Age-group-based subdivision of samples used for anti-SARS-CoV-2 IgG and anti-citrate synthase IgG/M screening.

Age Group	Number of Vaccinated Samples	Total Number of Vaccinated Samples (Vaccinated + Unvaccinated)
11–20 y	8	8
21–30 y	21	21
31–40 y	30	50
41–50 y	26	47
51–60 y	50	67
61–70 y	50	61
71–80 y	30	49
81–90 y	22	27
TOTAL	237	330

**Table 3:** Vaccine-regimen-based, numerical subdivision of samples used for anti-SARS-CoV-2 IgG and anti-citrate synthase IgG/M screening.

Vaccination	Number of Samples	Ratio of All Vaccinated Individuals
mRNA	170	72%
mRNA + adenoviral vector	42	18%
Adenoviral vector	25	10%
Unvaccinated (control)	93	-
Vaccinated TOTAL	237	100%
TOTAL	330	-

## Data Availability

Research data and investigation results are available upon request.

## Supplementary Materials

The following supporting information can be downloaded at: https://www.mdpi.com/article/10.3390/ijms241914961/s1.

## Abbreviations

AAb	Autoantibody
AU	arbitrary unit (in-house ELISAs)
CS	citrate synthase
ELISA	Enzyme-linked immunosorbent assay
HIT	Herd immunity threshold
IgG	Immunoglobulin, G isotype
IgM	Immunoglobulin, M isotype
IU	international units
MMR	measles, mumps, rubella
mRNA	messenger ribonucleic acid
n	number of samples
nAAb	Natural autoantibody
OD	Optical density
PVA	polyvinyl alchol
RBD	Receptor Binding Domain
RT	room temperature
RU	relative unit (Euroimmun ELISAs); quantitative measurement entity in linear correlation with the “First WHO International Standard for SARS-CoV-2”
S1	S1 Subunit of the SARS-CoV-2 Spike Protein
SARS-CoV-2	severe acute respiratory syndrome coronavirus 2
WB	washing buffer
y	years of age
