# Supplementary material for: Following Natural Autoantibodies: Further Immunoserological Evidence Regarding Their Silent Plasticity and Engagement in Immune Activation
Source: Int J Mol Sci. 2023 Oct 6;24(19):14961. doi: 10.3390/ijms241914961 (PMC10573785; doi:10.3390/ijms241914961)
Supplement: Supplementary file 1 [file ijms-24-14961-s001.zip › ijms-2609975-supplementary.pdf]

Supplementary Materials

Supplementary Table S1. (for Figure 1). Sample numbers used for MMR seropositivity ratio (%) calculation. Samples with extrapolated results (value ≤ limit of quantification or value ≥ upper limit of detection) were excluded from the statistical evaluation, hence the varying sample numbers per compound of interest.

Age Group	Total Number of Samples/Age Group	Number of Seropositive Samples/Antigen		
		Measles	Mumps	Rubella
20-30 y	143	126	123	132
31-40 y	279	211	235	242
41-50 y	359	278	285	299
51-60 y	307	225	238	265
61-70 y	291	257	248	273
71-80 y	253	236	214	226
81-90 y	107	98	95	96
TOTAL	1739	1431	1438	1533

Supplementary Table 2. (for Figure 3) Sample numbers used for anti-SARS-CoV-2 seropositivity ratio (%) calculation, only from vaccinated individuals. From the serum bank, a representative number of samples for each age group was selected to be measured with the commercial (Eruoimmun Quantivac) ELISA assay as well as with our in-house anti-CS assay.

Age Group	Number of Samples	Number of Seropositive Samples Cut-Off of the Equivocal Range: ≥ 8 RU/mL	Seropositivity Ratio % Cut-Off of the Equivocal Range: ≥ 8 RU/mL	Number of Seropositive Samples Cut-Off of the Positive Range: ≥ 11 RU/mL	Seropositivity Ratio % Cut-Off of the Positive Range: ≥ 11 RU/mL
21-30 y	21	21	100.00	21	100.00
31-40 y	30	30	100.00	30	100.00
41-50 y	26	24	92.31	24	92.31
51-60 y	50	49	98.00	48	96.00
61-70 y	50	48	96.00	46	92.00
71-80 y	30	24	80.00	24	80.00
81-90 y	22	22	100.00	22	100.00
TOTAL	237	218	91.98	215	90.72
